# Overproduction of pro-transglutaminase from *Streptomyces hygroscopicus* in *Yarrowia lipolytica* and its biochemical characterization

**DOI:** 10.1186/s12896-015-0193-1

**Published:** 2015-08-14

**Authors:** Song Liu, Dan Wan, Miao Wang, Catherine Madzak, Guocheng Du, Jian Chen

**Affiliations:** Key Laboratory of Industrial Biotechnology, Ministry of Education, Jiangnan University, Lihu Avenue, Wuxi, China; School of Food Science and Technology, Jiangnan University, Lihu Avenue, Wuxi, China; National Engineering Laboratory for Cereal Fermentation Technology, Jiangnan University, Lihu Avenue, Wuxi, China; Key Laboratory of Carbohydrate Chemistry and Biotechnology, Ministry of Education, Jiangnan University, Lihu Avenue, Wuxi, China; INRA, UMR1319 Micalis, Domaine de Vilvert, F-78352 Jouy-en-Josas, France; Present address: INRA, UMR 782 Génie et Microbiologie des Procédés Alimentaires, AgroParisTech campus, CBAI, F-78850, Thiverval-Grignon, France

**Keywords:** Transglutaminase, *Yarrowia lipolytica*, Secretory expression, Enzymatic property

## Abstract

**Background:**

Transglutaminases (TGase), synthesized as a zymogen (pro-TGase) in *Streptomyces* sp., are important enzymes in food industry. Due to the important applications of TGase in food industry, obtaining robust and food-safe TGase-producing strains has attracted much attention during the past decade. In this study, *Streptomyces hygroscopicus* pro-TGase was efficiently expressed and secreted by a food-grade host, *Yarrowia lipolytica*, without antibiotic markers.

**Results:**

The pro-TGase gene was cloned into integrative vectors pINA1296 (monocopy) and pINA1297 (multicopy), and was used to transform the *Y. lipolytica* Po1g or Po1h strain, respectively. Expression was driven by a recombinant hp4d promoter and secretion obtained using a *XPR2* pre-sequence as a signal peptide. The highest yield of extracellular pro-TGase produced by the recombinant Po1h strain corresponded to 5.3 U/mL of TGase, a level 8.8 fold higher than that obtained using the recombinant Po1g strain. Asparagines in two potential Asn-linked glycosylation sites (Asn160 and Asn355) from pro-TGase were mutated to glutamine individually or simultaneously, yielding the deglycosylated variants N160Q, N355Q, and N160Q/N355Q. The activities of N160Q, N355Q and N160Q/N355Q constructs were respectively 5.3 U/mL, 7.8 U/mL, and 3.0 U/mL, equivalent to 100 %, 147 %, and 57 % of that from wild-type pro-TGase. The TGase yield of N355Q variant was raised to 35.3 U/mL of by using a glycerol feeding strategy in a 3 L fermenter. The optimal pH and temperature of the activated pro-TGase, and of its deglycosylated variants, were in the range of 5.0-6.0 pH and 40-45 °C, respectively. The half-life of the recombinant wild-type pro-TGase at 37 °C reached 34.0 min, and those of the variants were from 24.2 min to 11.5 min. In contrast to the wild-type pro-TGase, all of the variants had decreased specific activities, and both the *K*_m_ and *k*_cat_ values of the variants decreased accordingly.

**Conclusions:**

This study constitutes the first report of the heterologous expression of a pro-TGase in *Y. lipolytica*, and provides new possibilities for the efficient production of TGases used in food processing.

## Background

Transglutaminases (EC 2.3.2.13, TGases) catalyze crosslinking between γ-carboxyamide groups in glutamine residues (acyl donors) and a variety of primary amines (acyl acceptors) in many proteins [1]. Due to this unique protein bonding reaction, TGases have been used to improve the functional properties of protein-based food ingredients, such as their texture, stability, and water binding capacity [1, 2]. TGases are widely distributed in various organisms, including mammals [3], plants [4] and microorganisms [5]. Among the TGase enzymes, those from *Streptomyces* sp. are particularly advantageous for industrial applications because of their Ca^2+^-independence, higher reaction rate, broad substrate specificity for acyl donors and smaller molecular size [1]. To date, TGases used in food industry are mainly obtained from *Streptomyces* genus.

To improve yields, various heterologous expression hosts have been examined for the production of *Streptomyces* TGases, such as *Escherichia coli* [6], *Streptomyces lividans* [7], *Corynebacterium glutamicum* [8] and methylotrophic yeasts [9]. *Streptomyces* TGase is naturally synthesized as a zymogen (pro-TGase), which is then processed to produce active enzyme by removal of its N-terminal pro-peptide [5]. As production of active TGase leads to cell death by cross-linking host proteins [10], *Streptomyces* TGase is usually expressed in the form of pro-TGase in heterologous hosts, and the resulting pro-enzyme is converted into active TGase either by *in vitro* addition of activation protease [11] or by co-expressing the protease [12]. So far, some of these recombinant strains have achieved high-level expression of pro-TGase, such as recombinant *C. glutamicum* YDK010 (ATCC6872) which secreted 881 mg/L of pro-TGase [8]. However, these recombinant strains contain antibiotic resistance markers, which imply a risk of transferring antibiotic resistance to the human intestinal microflora [13]. Based on food safety standards, there is a need to consider new expression systems without antibiotic resistance markers for TGase production.

Recently, *Yarrowia lipolytica* has been developed as a suitable expression host for heterologous proteins, including some food processing enzymes [14]. Several characteristics contribute to its suitability: (i) it is considered to be a GRAS (Generally Regarded As Safe) microorganism by the Food and Drug Administration (FDA, USA); (ii) the use of auto-cloning vectors, from which a “yeast cassette” can be targeted non-homologously into the genome allows constructing production strains devoid of antibiotic resistance genes; (iii) the Po1 series of recipient strains have been deleted for extracellular proteases; (iv) the widely used recombinant hp4d promoter is able to drive strong expression in virtually any medium, almost independent of environmental conditions; (v) the defective *ura3d4* selection marker (multicopy vectors) allows amplifying the copy number and, concomitantly, the expression of the gene of interest [14, 15]. In addition, a high-throughput screening system based on *Y. lipolytica* has been developed, which will facilitate genetic engineering or the directed evolution of enzymes [16, 17].

The objective of this study was to construct a recombinant *Y. lipolytica* strain that could efficiently secrete pro-TGase from *Streptomyces hygroscopicus* WSH03-13. At first, the productivities of a monocopy vector and a multicopy vector expressing *S. hygroscopicus* pro-TGase were examined in *Y. lipolytica*. Then, asparagines in two predicted Asn-linked glycosylation sites (Asn160 and Asn355) of pro-TGase were mutated to glutamines, individually or simultaneously, in order to tentatively improve TGase properties. The productivity of a selected recombinant *Y. lipolytica* strain was also tested in jar fermenter. Finally, the catalytic properties of TGases derived from glycosylated or non-glycosylated pro-TGases were analyzed and compared.

## Results

### Heterologous expression of pro-TGase

**Fig. 1:**
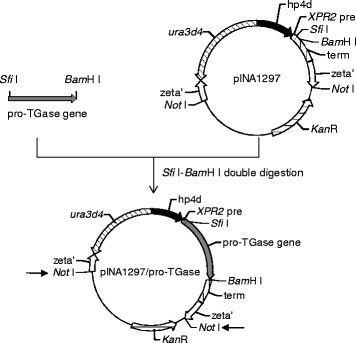
Construction of the recombinant expression vector pINA1297/pro-TGase. The hp4d promoter is indicated as a black arrow; the *XPR2* signal sequence is indicated by a white box; the terminator is indicated by a box filled with vertical lines; the genes for selecting in *Y. lipolytica* or *E. coli* are indicated by slashed arrows; the zeta elements are indicated as two white arrows. The pro-TGase gene is inserted between the *Sfi* I and *Bam*H I sites. The linearization sites allowing to liberate the “yeast cassette” are indicated by small arrows

The *S. hygroscopicus* pro-TGase gene was inserted downstream of a secretion signal (*XPR2* pre-sequence) in two integrative vectors, pINA1296 (a pBR322-based mono-copy vector) and pINA1297 (a zeta-based auto-cloning multi-copy vector) [14], yielding pINA1296/pro-TGase and pINA1297/pro-TGase, respectively (Fig. 1). Because pINA1296 and pINA1297 carry different selection markers and are based on different integration mechanisms [14], *Not* I-digested pINA1296/pro-TGase (linearized vector) and pINA1297/pro-TGase (purified “yeast cassette”) were used to transform the *Y. lipolytica* Po1g (Leu^−^, containing a pBR322 docking platform) and Po1h (Ura^−^) strains, respectively [14].

**Fig. 2:**
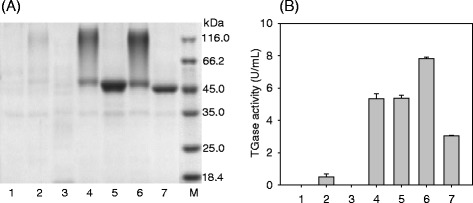
Pro-TGase production by yeast transformants. **a** SDS-PAGE analysis of pro-TGase secretion by recombinant *Y. lipolytica* strains carrying the following constructs. **b** Extracellular yield of pro-TGase and its variants produced by the corresponding recombinant *Y. lipolytica* strains. Labeling for both (**a**) and (**b**): 1: pINA1296, 2: wild-type pro-TGase (pINA1296/pro-TGase), 3: pINA1297, 4: wild-type pro-TGase (pINA1297/pro-TGase), 5: N160Q, 6: N355Q, 7: N160Q/N355Q, M: protein marker. All of the transformants were cultivated at 28 °C and 200 rpm for 5 days using a modified PPB medium; the TGase activities of pro-TGase and its variants were obtained following *in vitro* activation (see “Methods”). Each value represents the mean of three independent measurements

**Fig. 3:**
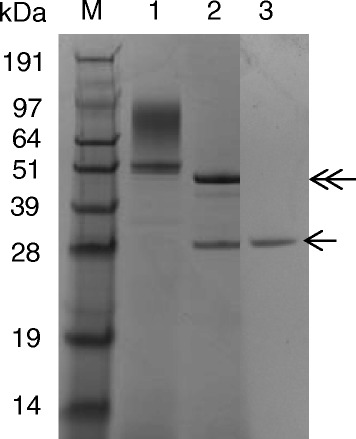
Endo H treatment of pro-TGase produced by *Y. lipolytica* carrying pINA1297/pro-TGase. M: protein marker, 1: wild-type pro-TGase (pINA1297/pro-TGase), 2: wild-type pro-TGase (pINA1297/pro-TGase) treated with Endo H, 3: Endo H solution. The Endo H treatment method is described in the “Methods” section. The position of Endo H and deglycosylated pro-TGase are indicated by a single-headed arrow and a double-headed arrow, respectively

The recombinant strains were cultivated in PPB medium at 28 °C for 120 h. As shown in Fig. 2a, the pro-TGase band (43.6 kDa) was not clearly visible in the culture supernatant of either of the recombinant strains. However, a smear in the range of 48-120 kDa was observed in the culture supernatant of cells carrying pINA1297/pro-TGase (Fig. 2a). Endo H treatment of the culture supernatant of cells carrying pINA1297/pro-TGase eliminated the high molecular weight smear (Fig. 3). Following the addition of dispase to the culture supernatants, cells harboring pINA1297/pro-TGase yielded 5.3 U/mL of TGase activity, a level that was 8.8-fold higher than that produced by cells carrying pINA1296/pro-TGase (Fig. 2b). Control samples did not exhibit TGase activity under the same conditions (Fig. 2b).

### Effect of Asn-linked glycosylation on the production of pro-TGase

Two potential Asn-linked glycosylation sites, corresponding to Asn160 and Asn355, were predicted by CBS NetNGlyc 1.0 Server. Based on the pINA1297/pro-TGase, Asn160 and Asn355 were mutated to glutamine, yielding pro-TGase derivatives N160Q and N355Q, respectively; the simultaneous mutation of the two sites produced N160Q/N355Q. All of these variant versions of pINA1297/pro-TGase were expressed in *Y. lipolytica* Po1h. As shown by SDS-PAGE, N355Q displayed a smeared band similar to that observed for the wild-type pro-TGase (Fig. 2a). In contrast, both N160Q and N160Q/N355Q exhibited a sharp band, with slightly different migration positions (Fig. 2a). Following activation by dispase, the activities of N160Q, N355Q and N160Q/N355Q constructs were 5.3 U/mL, 7.8 U/mL and 3.0 U/mL, respectively, which are equivalent to 100 %, 147 %, and 57 % of that from the wild-type pro-TGase (Fig. 2a).

### Production of N355Q in 3 L fermenter

**Fig. 4:**
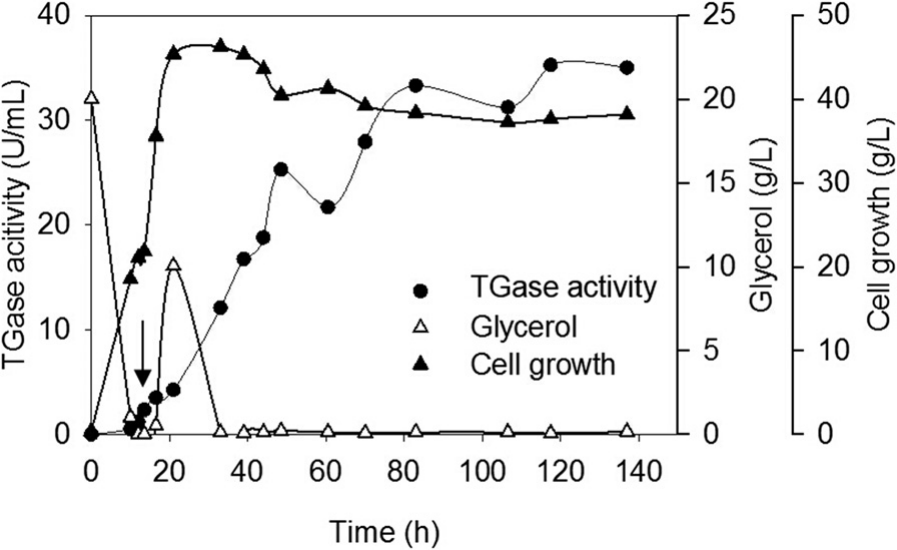
Production of N355Q in 3 L fermenter. Overnight culture of the recombinant *Y. lipolytica* expressing N355Q in liquid YPD medium (100 mL) was inoculated into 1 L of modified PPB medium and cultivated for 140 hours. See Methods for detailed fermenter operating conditions. The arrow indicates the starting point for glycerol feeding. Each value represents the mean of three independent measurements

The recombinant strain expressing N355Q was subjected to a cultivation test in a jar fermenter. The fermentation condition is described in the “Methods” section. As shown in Fig. 4, the glycerol in the initial medium was exhausted after 12 h of fermentation, and a glycerol solution (50 %, w/v) was then fed at a rate of 7.5 g/h for 6.5 h. Then, the added glycerol was quickly used up in the following 21 h. Dry cell concentration grew quickly during the first 21 h and decreased slowly afterwards (Fig. 4). As indicated by TGase activity, the culture supernatant accumulated little pro-TGase variant N355Q at the beginning of the fermentation (Fig. 4). However, this accumulation increased significantly after starting glycerol feeding. The yield of pro-TGase reached its highest value (35.3 U/mL of corresponding TGase) at 117.5 h.

### Biochemical characterization of activated pro-TGase and its variants

**Fig. 5:**
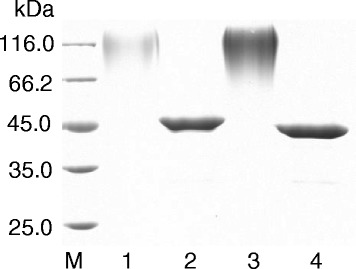
SDS-PAGE analysis of purified pro-TGase and its variants. Culture supernatants containing pro-TGase or its variants were obtained by centrifugation and purified by ion exchange chromatography on a Fractogel EMD SO^3−^ (see “Methods”). M: protein marker, 1: wild-type pro-TGase (pINA1297/pro-TGase), 2: N160Q, 3: N355Q, 4: N160Q/N355Q

The recombinant pro-TGase and its variants were purified by ion exchange chromatography (see “Methods”). A single protein band was obtained, for each sample, from the finally concentrated elutes (Fig. 5). Following activation by dispase, the biochemical characteristics of the recombinant pro-TGase and its variants were examined.

**Fig. 6:**
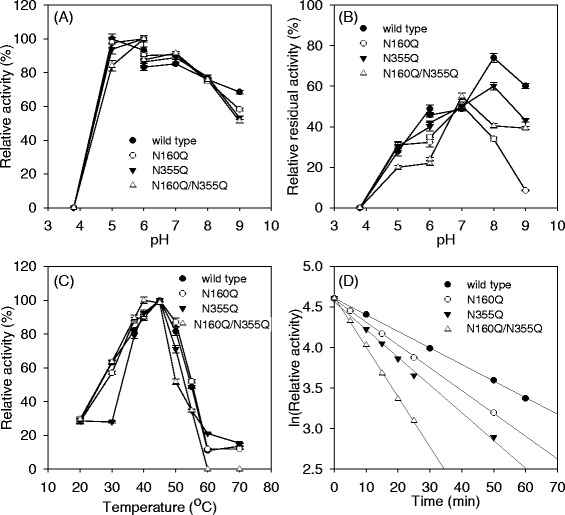
Enzymatic properties of activated pro-TGase and its variants. **a** The effects of pH on TGase activity. **b** The effects of pH on TGase stability. **c** The effects of temperature on TGase activity. **d** The effects of temperature on TGase stability at 37 °C. The pH of the reaction mixture was adjusted with 50 mM acetate buffer (pH 3.7–6.0) and 50 mM Tris–HCl buffer (pH 6.0–9.0). The tests for the effects of pH on TGase activity and stability were performed at 37 °C. The effects of temperature on TGase activity was determined at pH 6.0. The thermal stability was examined by incubating enzyme solution at different temperatures for 0-60 min, and measuring residual activities at 37 °C and pH 6.0. Each value represents the mean of three independent measurements

All activated pro-TGase variants exhibited an optimal pH activity profile (pH 5.0–7.0) similar to that of the recombinant wild-type TGase (Fig. 6a) but more acidic than that of TGase naturally secreted by *S. hygroscopicus* (pH 6.0-7.0) [18]. When incubated at different pH levels, the activated pro-TGase and N355Q retained their highest residual activities at a pH of 8.0, while N160Q and N160Q/N355Q did so at pH7.0 (Fig. 6b). The activated N160Q/N355Q had its highest activity at 40 °C, and the other activated zymogens displayed their highest activities at 45 °C (Fig. 6c). All of the activated zymogens retained approximately 80 % of the TGase activity after incubation at 20 °C for 30 min and were approximately inactivated after the same time at 50 °C (data not shown). When treated at 37 °C, the decline rates of ln (relative enzyme activity) increased according to the following sequence: activated pro-TGase, N160Q, N355Q, and N160Q/N355Q (Fig. 6d), and the corresponding *t*_1/2_ values at 37 °C decreased accordingly (Table 1). As shown in Table 1, the specific activities of all activated variants decreased in contrast to the wild-type TGase. The *K*_m_ values of activated variants were lower than that of the wild-type TGase (Table 1), suggesting an increased substrate affinity for the variants. All activated variants exhibited a remarkable decrease in *k*_cat_, which finally lowered their specific activities (Table 1).

**Table 1:** Enzymatic properties of activated pro-TGase and its variants

	Wild type	N160Q	N355Q	N160Q/N355Q
*t*_1/2_ (37 °C) (min)	34.0 ± 0.2	24.2 ± 0.1	19.2 ± 0.2	11.5 ± 0.2
Specific activity (U/mg)	16.6 ± 0.2	14.7 ± 0.5	13.3 ± 0.3	10.4 ± 1.3
*K*_m_ (mmol/L)	59.5 ± 2.3	47.0 ± 1.5	54.7 ± 2.1	48.4 ± 2.4
*k*_cat_ (s^−1^)	31.5 ± 1.7	25.3 ± 2.2	23.8 ± 3.2	16.6 ± 1.7

Note: *t*_1/2_ (37 °C) was determined at an enzyme concentration of approximately 0.5 mg/mL for each enzyme; the specific activity measurements were performed using N-CBZ-Gln-Gly as the substrate (30 mmol/L); and kinetic constants were obtained at substrate (N-CBZ-Gln-Gly) concentrations ranging from 6 mmol/L to 30 mmol/L

## Discussion

Due to the important applications of TGase in food industry, obtaining robust and food-safe TGase-producing strains has attracted much attention during the past decade (Table 2). However, the relatively low production and the introduction of antibiotic resistance markers has limited the application of these recombinant strains for commercial scale (Table 2). In this study, *S. hygroscopicus* pro-TGase was successfully expressed in a food-grade host strain *Y. lipolytica* Po1h using pINA1297 vector (Figs. 1 and 2). By employing a glycerol feeding strategy in a 3 L fermenter, the yield of a pro-TGase variant (N355Q) reached 35.3 U/mL (Fig. 4), which is the highest yield of pro-TGase reported to date (Table 2). Before being transformation into *Y. lipolytica* Po1h, the kanamycin resistance gene (Kan^R^) was removed from the pINA1297 derivatives expressing pro-TGase (Fig. 1), eliminating the potential risk of transfer of the antibiotic resistance to the human intestinal microflora. Overexpression of pro-TGase in the food-grade host *Y. lipolytica* strain without antibiotic resistance marker provides new possibilities for efficient production of TGases used in food processing.

**Table 2:** Extracellular expression of *Streptomyces* TGases in heterologous systems

Reference	Gene resource	Host strain	Antibiotic resistance for selection	Yield (U/mL)	Productivity (U/mL/h)
Yurimoto et al. [9]	*Streptoverticillium mobaraense* S-8112	*C. boidinii* BUL	Amp^R^	1.8	0.02
Lin et al. [32]	*S. platensis* M5218	*S. lividans* JT46	Tsr^R^	2.2	0.03
Liu et al. [33]	*S. fradiae*	*S. fradiae*	Apr^R^	3.2	0.07
Date et al. [8]	*Sv. mobaraense*	*C. glutamicum* YDK010	Kan^R^	16.3	0.41
Noda et al. [34]	*Sv. cinnamoneum*	*S. lividans* 1326	Amp^R^/Tsr^R^	2.8	0.02
Liu et al. [27]	*S. hygroscopicus*	*E. coli* BL21(DE3)	Amp^R^	4.5	0.11
This work	*S. hygroscopicus*	*Y. lipolytica* Po1h	none (selection by Ura^−^ auxotrophy complementation)	35.3	0.30

Abbreviation: Amp^R^, ampicillin resistance; Tsr^R^, thiostrepton resistance; Apr^R^, apramycin resistance; Kan^R^, kanamycin resistance; Ura^−^, uracil auxotrophy

In the present study, cells carrying pINA1297/pro-TGase showed smeared high molecular weight bands (48–120 kDa) (Fig. 2a), which were transformed into a single pro-TGase band by Endo H treatment (Fig. 3). These results indicate that N-linked glycosylation of pro-TGase occurred during its expression in *Y. lipolytica*. To identify the sites glycosylated by *Y. lipolytica*, variants of pro-TGase carrying mutated N-linked glycosylation sites were constructed. Protein deglycosylation is typically achieved by site-directed mutagenesis of the asparagine (Asn, N) residue of Asn-Xaa-Ser/Thr consensus sequences to the structurally related glutamine (Gln, Q) residue, or to other amino acids such as alanine (Ala, A) [19]. In our case, we have substituted asparagines in two putative N-glycosylation sites (Asn160 and Asn355) with glutamine both individually and simultaneously. The substitution at Asn355 (N355Q) did not eliminate the smeared bands, while the mutation at Asn160, alone (N160Q) or together with Asn355 (N160Q/N355Q), led to a sharp pro-TGase band (Fig. 2a). To be noted, the molecular weight of N160Q was bigger than that of N160Q/N355Q which was similar to the theoretical molecular weight of *S. hygroscopicus* pro-TGase (43.5 kDa) (Fig. 2a). Generally, smeared bands suggest the presence of heterogeneous glycoforms, whereas sharp bands indicate a homogenous glycoform [20]. Thus, heterogeneous glycosylation seems to occur at Asn 160 and homogenous glycosylation at Asn 355 in pro-TGase produced in *Y. lipolytica*.

In comparison with wild-type pro-TGase, deglycosylation at Asn160 alone (N160Q), or together with that at Asn355, exerted no effect or a negative effect on the secretion of pro-TGase (Fig. 2). Unexpectedly, deglycosylation at Asn355 (N355Q) had superior extracellular yield in *Y. lipolytica* in contrast to the wild-type pro-TGase (Fig. 2). Although how glycosylation affects the secretion level is still not fully clarified, it is generally recognized that glycosylation benefits protein secretion by mitigating aggregation and hydrolysis in yeasts [21]. To the best of our knowledge, no previous report has described the positive effects of deglycosylation on protein secretion in yeasts [22, 23]. However, deglycosylation by itself may not account for the observed increase in pro-TGase secretion by *Y. lipolytica*. Since pINA1297 vector has a random integration mechanism into *Y. lipolytica* genome [14], transformants are usually different in terms of the integration loci and copy number, which is expected to affect protein expression level [16]. Thus, the selected transformant expressing N355Q might be the result of the integration(s) at a locus (loci), leading to higher expression and/or of higher copy number than transformants expressing the wild-type pro-TGase.

The wild-type pro-TGase and its variants (N160Q, N355Q, and N160Q/N355Q) had different catalytic properties. Although the wild-type pro-TGase shared a similar pH profile with its deglycosylated variants (Fig. 6a), the former was more stable in alkaline conditions than the latters (Fig. 6b). This might be explained by the fact that the Asn-glycans in glycosylated proteins could cause a local perturbation of charged amino acids, resulting into a shift in the protonation state of the carboxylate moiety [24]. The optimal temperature of N160Q/N355Q shifted from 45 °C, which was the optimal temperature of TGase naturally produced by *S. hygroscopicus* [18], to 40 °C (Fig. 6c). The thermal stability of wild-type pro-TGase was higher than that of the deglycosylated variants (Fig. 6d and Table 1). Thermal stabilization of proteins by glycosylation has been reported in many cases, such as for β-d-glucuronidase [24] and tannase [25]. The enhanced thermal stability of proteins by glycosylation is generally attributed to destabilization of their unfolded states [26]. In contrast to wild-type pro-TGase, the variants had reduced specific activities. Although the *K*_m_ values of the variants decreased, their *k*_cat_ values also decreased accordingly compared with the wild-type enzyme (Table 1). Similar negative effects of deglycosylation on the specific activities and *K*_m_ values were also observed in the expression of β-glucosidase in *P. pastoris* [20]. The exact mechanism of glycosylation regulating the *K*_m_ and *k*_cat_ values may be elucidated by crystal structure analysis of the recombinant pro-TGases.

## Methods

### Plasmids and strains

The pBB1-1020 vector, encoding the pro-TGase gene (GenBank ID: HM231108) from *S. hygroscopicus* WSH03-13, was described in a previous study [27]. The *E. coli* JM109 strain (Novagen, USA) was used for vectors construction. *Y. lipolytica* Po1g (*MatA, leu2-270, ura3-302::URA3, xpr2-322, axp1-2;* Leu^−^, △AEP, △AXP, Suc^+^, pBR322) and Po1h (*MatA, ura3-302, xpr2-322, axp1-2;* Ura^−^, △AEP, △AXP, Suc^+^) strains were used as expression hosts [14]. pINA1296 (a pBR322-based mono-copy integrative vector) and pINA1297 (an auto-cloning multi-copy integrative vector) were used as expression vectors in *Y. lipolytica* Po1g and Po1h strains [14], respectively.

### Expression vector construction

To construct the plasmid expressing pro-TGase, the pro-TGase gene was amplified from pBB1-1020 using the primers pair 1296P1/1296P2 and 297P1/1297P2 (Table 3). PCR amplification conditions were as follows: initial denaturation at 98 °C for 3 min, followed by 30 amplification cycles, each consisting of 98 °C for 30 s, 58 °C for 30 s, and 72 °C for 80s. The PCR fragments were double-digested with either *Hind* III-*Kpn* I or *Sfi* I -*Bam*H I. The resulting pro-TGase fragments were cloned into the *Hind* III-*Kpn* I sites of pINA1296 to generate pINA1296/pro-TGase and into the *Sfi* I-*Bam*H I sites of pINA1297 to generate pINA1297/pro-TGase (Fig. 1).

**Table 3:** Primers used in this study

Primer	Sequence (5′-3′)
1296P1	CCCAAGCTTGCCAGCGGCGGCGACG (underlined bases correspond to *Hind* III site)
1296P2	CGGGGTACCTTACGACCAGCCCTGCTTCACCTC (underlined bases correspond to *Kpn* I site)
1297P1	TTGGGCCGTTCTGGCCGCCAGCGGCGGCGACG (underlined bases correspond to *Sfi* I site)
1297P2	CGCGGATCCTTACGACCAGCCCTGCTTCACCTC (underlined bases correspond to *Bam*H I site)
7N160Q1	CCCCAGGAGACGCAAGCCGAGTTT (underlined bases encode Gln)
7N160Q2	GCGTGGCCTGCTGTTTTCCAGGTC
7N355Q1	CGGCAGTGGTCTGCCGGGTA (underlined bases encode Gln)
7N355Q2	GAACTTGCTCTCGTATACGTTCATGGG

### Mutation of the Asn-linked glycosylation sites

The Asn-linked glycosylation sites of pro-TGase were predicted by CBS NetNGlyc 1.0 Server (http://www.cbs.dtu.dk/services/NetNGlyc/). Site-directed mutagenesis of pINA1297/pro-TGase was conducted by a PCR method with mutagenic primers pairs, using the MutanBEST Kit (TaKaRa, Dalian, China). The two primers pairs 7N160Q1/7N160Q2 and 7N355Q1/7N355Q2 were designed to mutate Asn160 and Asn355, respectively, of the pro-TGase to Gln (Table 3).

### Transformation of *Y. lipolytica*

pINA1296/pro-TGase and pINA1297/pro-TGase vectors were digested with *Not* I (Fig. 1) and the resulting DNA fragments carrying the pro-TGase gene were recovered using the Agarose Gel DNA Purification Kit Ver.2.0 (TaKaRa, Dalian, China). *Not* I digestion linearizes the pINA1296/pro-TGase vector in the pBR322 region, allowing targeting its integration into the pBR322 docking platform of the Po1g strain [14]. In contrast, in the pINA1297/pro-TGase vector, *Not* I digestion liberates a “yeast cassette” devoid of bacterial DNA, bordered by zeta sequences and containing the *ura3d4* defective selection marker. Zeta sequences are LTRs (long terminal repeats) of Ylt1 retrotransposon and have the property to promote non-homologous integration into the genome of Po1h strain [14]. The defective *ura3d4* allele is required in multiple copies to complement the auxotrophy of the Po1h host, which leads to an amplification of the copy number of “yeast cassette” (hp4d::*XPR2*pre::pro-TGase:: *XPR2*term) integrated into the host genome [14]. The method described by Xuan et al. [28] was used for the transformation of *Y. lipolytica*, with selection on YNB-N_5000_ (1.7 g/L yeast nitrogen base without amino acids and ammonium sulfate, 10 g/L glucose, and 5 g/L ammonium sulfate) minimal medium plates.

### Protein expression

*Y. lipolytica* transformants were grown on YPD plates (10 g/L yeast extract, 20 g/L polypeptone, 20 g/L glucose, and 20 g/L agar) at 28 °C for 2 days. Transformant colonies were inoculated into liquid YPD medium and cultivated at 28 °C and 200 rpm. Overnight cultures of the recombinant strains (2.5 mL) were inoculated into 25 mL of modified PPB medium, in 250 mL flasks, and cultivated at 28 °C and 200 rpm for 5 days. Modified PPB medium contained 20 g/L glucose, 1.32 g/L yeast extract, 1.32 g/L NH_4_Cl, 0.32 g/L KH_2_PO_4_, 0.24 g/L MgSO_4_·7H_2_O, and 0.033 g/L thiamine and was adjusted to a pH of 6.0 [29].

### Jar fermentation

Fermentation was performed in a 3 L jar fermenter (Biotron, South Korea). Overnight cultures of recombinant strains in liquid YPD medium (100 mL) were inoculated into 1 L of modified PPB medium and cultivated for 140 h. The jar fermenter operating conditions were as follows: agitation rate = 600 rpm; airflow rate = 2.0 vvm; and temperature = 28 °C. When the initial glycerol was exhausted, a glycerol solution (50 %, w/v) was fed at a rate of 7.5 g/h for 6.5 h. Residual glycerol was measured by high performance liquid chromatography (HPLC) assay using a Waters 600 HPLC system (Waters Corp., USA). The HPLC parameters were set as follows: C18 reverse phase column (Sugar Pak I, Waters); mobile phase: water; flow rate: 0.4 mL/min; column temperature: 85 °C; detector: differential refraction detector; sample size: 10 μL. The dry cell weight was determined to follow cell growth.

### Protein purification

Culture supernatants containing pro-TGase were obtained by centrifugation at 10,000 x g for 20 min and were then dialyzed against buffer A (20 mM NaAc-HAc, pH 5.0) for 24 h, using cellulose ester membranes. Next, enzyme solutions were purified by ion exchange chromatography (IEC) on a Fractogel EMD SO_3_^−^ (2.6 x 14 cm) equilibrated with buffer A. After eluting unbound proteins at a flow rate of 1 mL/min, a linear elution was performed using NaCl (from 0 to 0.8 mol/L) and fractions were collected for activity assay and sodium dodecyl sulfate polyacrylamide gel electrophoresis (SDS-PAGE) analysis.

### Pro-TGase activation and the TGase activity assay

Pro-TGase activation, using dispase (Worthington, NJ, USA), was performed as previously described [27]. TGase activity was measured using a colorimetric procedure in which N-α-carbobenzoxyl-glutaminyl-glycine (N-CBZ-Gln-Gly) (Sigma, Shanghai, China) was used as the substrate [6]. Forty microliters of substrate solution (30 mmol/L N-CBZ-Gln-Gly, 100 mmol/L hydroxylamine, 10 mmol/L glutathione, and 200 mmol/L Tris–HCl buffer at a pH of 6) was added to 100 μL of TGase solution to initiate enzymatic reaction. After 10 min, the reaction was stopped by the addition of 40 μL of terminator solution (1 mol/L HCl, 4 % (v/v) trichloroacetic acid, 2 % (m/v) FeCl_3_·6H_2_O) and the mixture was subjected to spectrophotometry analysis at 525 nm. A calibration curve was obtained using L-glutamic acid γ-monohydroxamate (Sigma, Shanghai, China). One unit of TGase was defined as the amount required to generate 1 μmol of L-glutamic acid γ-monohydroxamate per min at 37 °C. The results are the average of triplicate assays.

### Protein analysis

SDS-PAGE was performed on a 12 % running gel, and the resolved proteins were visualized by staining with Coomassie Brilliant Blue R250. Protein concentrations were measured using the Bradford method, with bovine serum albumin as the standard [30].

### Endo H treatment of Pro-TGase

Endo H (New England Biolabs, USA) was used to deglycosylate the secreted pro-TGase. The culture supernatant of *Y. lipolytica* expressing pro-TGase was denatured for 10 min at 100 °C, and then incubated with Endo H at 37 °C for 1 h according to the manufacturer’s instruction.

### Biochemical characteristics

The effect of pH on TGase activity was determined at 37 °C in various buffers: 50 mmol/L NaAC-HAC, pH 3.7-6.0; and 50 mmol/L Tris–HCl, pH 6.0-9.0. The pH stability was determined by incubating the enzyme solution at different pH levels (3.7 to 9.0), at 37 °C for 1 h, and measuring the residual TGase activity at pH 6.0 and 37 °C. The effect of temperature on TGase activity was determined at temperatures ranging from 20 to 70 °C, at pH 6.0. Thermal stability was examined by incubating the enzyme solution at different temperatures (20 °C, 37 °C, and 50 °C) for 0-60 min, and measuring the residual activities at 37 °C and pH 6.0. As described in our previous study [31], the semi-log. graph of ln (relative enzyme activity) versus time was drawn, and the half-life (*t*_1/2_) of TGase in minutes was then obtained. The kinetic parameters of TGase were examined under 50 mmol/L Tris–HCl buffer (pH6.0) using N-CBZ-Gln-Gly (6-30 mmol/L) as the substrate. The values of the Michaelis constant (*K*_m_) and maximum velocity (*k*_cat_) were determined from Lineweaver-Burk plots.

## Conclusions

In this study, *S. hygroscopicus* pro-TGase is successfully expressed by a food-grade host *Y. lipolytica*. We demonstrated that heterogeneous glycosylation and homogenous glycosylation occur at Asn 160 and Asn 355 of pro-TGase in *Y. lipolytica*, respectively. We also found that wild-type pro-TGase and its deglycosylated variants (N160Q, N355Q, and N160Q/N355Q) had different catalytic properties. In conclusion, this study provides new possibilities for the efficient production of TGases used in food processing.

## References

[1] Yokoyama K, Nio N, Kikuchi Y (2004). Properties and applications of microbial transglutaminase. Appl Microbiol Biotechnol.

[2] Dube M, Schafer C, Neidhart S, Carle R (2007). Texturisation and modification of vegetable proteins for food applications using microbial transglutaminase. Eur Food Res Technol.

[3] Nemes Z, Marekov LN, Fesus L, Steinert PM (1999). A novel function for transglutaminase 1: attachment of long-chain omega-hydroxyceramides to involucrin by ester bond formation. Proc Natl Acad Sci U S A.

[4] Di Sandro A, Del Duca S, Verderio E, Hargreaves AJ, Scarpellini A, Cai G (2010). An extracellular transglutaminase is required for apple pollen tube growth. Biochem J.

[5] Zotzel J, Keller P, Fuchsbauer HL (2003). Transglutaminase from *Streptomyces mobaraensis* is activated by an endogenous metalloprotease. Eur J Biochem.

[6] Liu S, Zhang DX, Wang M, Cui WJ, Chen KK, Du GC, et al. The order of expression is a key factor in the production of active transglutaminase in *Escherichia coli* by co-expression with its pro-peptide. Microbial Cell Factories. 2011;10. 10.1186/1475-2859-10-112 PMC3286405 22196373

[7] Noda S, Miyazaki T, Tanaka T, Chiaki O, Kondo A (2013). High-level production of mature active-form *Streptomyces mobaraensis* transglutaminase via pro-transglutaminase processing using *Streptomyces lividans* as a host. Biochem Eng J.

[8] Date M, Yokoyama K, Umezawa Y, Matsui H, Kikuchi Y (2004). High level expression of *Streptomyces mobaraensis* transglutaminase in *Corynebacterium glutamicum* using a chimeric pro-region from *Streptomyces cinnamoneus* transglutaminase. J Biotechnol.

[9] Yurimoto H, Yamane M, Kikuchi Y, Matsui H, Kato N, Sakai Y (2004). The pro-peptide of *Streptomyces mobaraensis* transglutaminase functions in cis and in trans to mediate efficient secretion of active enzyme from methylotropic yeasts. Biosci Biotechnol Biochem.

[10] Washizu K, Ando K, Koikeda S, Hirose S, Matsuura A, Takagi H (1994). Molecular-cloning of the gene for microbial transglutaminase from *Streptoverticillium* and its expression in *Streptomyces-lividans*. Biosci Biotechnol Biochem.

[11] Yang HL, Pan L, Lin Y (2009). Purification and on-column activation of a recombinant histidine-tagged pro-transglutaminase after soluble expression in *Escherichia coli*. Biosci Biotechnol Biochem.

[12] Kikuchi Y, Date M, Yokoyama K, Umezawa Y, Matsui H (2003). Secretion of active-form *Streptoverticillium mobaraense* transglutaminase by *Corynebacterium glutamicum*: Processing of the pro-transglutaminase by a cosecreted subtilisin-like protease from *Streptomyces albogriseolus*. Appl Environ Microbiol.

[13] Peterbauer C, Maischberger T, Haltrich D (2011). Food-grade gene expression in lactic acid bacteria. Biotechnol J.

[14] Madzak C, Gaillardin C, Beckerich JM (2004). Heterologous protein expression and secretion in the non-conventional yeast *Yarrowia lipolytica*: a review. J Biotechnol.

[15] Groenewald M, Boekhout T, Neuveglise C, Gaillardin C, van Dijck PW, Wyss M (2014). *Yarrowia lipolytica*: safety assessment of an oleaginous yeast with a great industrial potential. Crit Rev Microbiol.

[16] Cambon E, Piamtongkam R, Bordes F, Duquesne S, Laguerre S, Nicaud JM (2010). A new *Yarrowia lipolytica* expression system: An efficient tool for rapid and reliable kinetic analysis of improved enzymes. Enzyme Microb Technol.

[17] Bordes F, Fudalej F, Dossat V, Nicaud JM, Marty A (2007). A new recombinant protein expression system for high-throughput screening in the yeast *Yarrowia lipolytica*. J Microbiol Methods.

[18] Cui L, Du G, Zhang D, Liu H, Chen J (2007). Purification and characterization of transglutaminase from a newly isolated *Streptomyces hygroscopicus*. Food Chem.

[19] Skropeta D (2009). The effect of individual N-glycans on enzyme activity. Bioorg Med Chem.

[20] Wei W, Chen L, Zou G, Wang Q, Yan X, Zhang J (2013). N‐glycosylation affects the proper folding, enzymatic characteristics and production of a fungal β-glucosidase. Biotechnol Bioeng.

[21] Hou J, Tyo KE, Liu Z, Petranovic D, Nielsen J (2012). Metabolic engineering of recombinant protein secretion by *Saccharomyces cerevisiae*. FEMS Yeast Res.

[22] Jolivet P, Bordes F, Fudalej F, Cancino M, Vignaud C, Dossat V (2007). Analysis of *Yarrowia lipolytica* extracellular lipase Lip2p glycosylation. FEMS Yeast Res.

[23] Parthasarathy R, Subramanian S, Boder ET, Discher DE (2006). Post-translational regulation of expression and conformation of an immunoglobulin domain in yeast surface display. Biotechnol Bioeng.

[24] Zou S, Xie L, Liu Y, Kaleem I, Zhang G, Li C (2012). N-linked glycosylation influences on the catalytic and biochemical properties of *Penicillium purpurogenum* β-d-glucuronidase. J Biotechnol.

[25] Mizuno T, Shiono Y, Koseki T (2014). Biochemical characterization of *Aspergillus oryzae* native tannase and the recombinant enzyme expressed in *Pichia pastoris*. J Biosci Bioeng.

[26] Shental-Bechor D, Levy Y (2008). Effect of glycosylation on protein folding: A dose book at thermodynamic stabilization. Proc Natl Acad Sci USA.

[27] Liu S, Zhang DX, Wang M, Cui WJ, Chen KK, Liu Y (2011). The pro-region of *Streptomyces hygroscopicus* transglutaminase affects its secretion by *Escherichia coli*. FEMS Microbiol Lett.

[28] Xuan J-W, Fournier P, Gaillardin C (1988). Cloning of the LYS5 gene encoding saccharopine dehydrogenase from the yeast *Yarrowia lipolytica* by target integration. Curr Genet.

[29] Jolivalt C, Madzak C, Brault A, Caminade E, Malosse C, Mougin C (2005). Expression of laccase IIIb from the white-rot fungus *Trametes versicolor* in the yeast *Yarrowia lipolytica* for environmental applications. Appl Microbiol Biotechnol.

[30] Bradford MM (1976). A rapid and sensitive method for the quantitation of microgram quantities of protein utilizing the principle of protein-dye binding. Anal Biochem.

[31] Xu Z, Liu S, Lu X, Rao S, Kang Z, Li J, et al. Thermal inactivation of a recombinant lipoxygenase from Pseudomonas aeruginosa BBE in the absence and presence of additives. J Sci Food Agric. 2014;94(9):1753–57. 10.1002/jsfa.6487 24272925

[32] Lin Y-S, Chao M-L, Liu C-H, Tseng M, Chu W-S (2006). Cloning of the gene coding for transglutaminase from *Streptomyces platensis* and its expression in *Streptomyces lividans*. Process Biochem.

[33] Liu X, Yang X, Xie F, Qian S (2006). Cloning of transglutaminase gene from *Streptomyces fradiae* and its enhanced expression in the original strain. Biotechnol Lett.

[34] Noda S, Ito Y, Shimizu N, Tanaka T, Ogino C, Kondo A (2010). Over-production of various secretory-form proteins in *Streptomyces lividans*. Protein Expression Purif.

